# Investigating the role of symptom valorisation in tuberculosis patient delay in urban areas in Portugal

**DOI:** 10.1186/s12889-023-17319-7

**Published:** 2023-12-05

**Authors:** Margarida de Morais, Sofia Sousa, Jéssica Marques, Marta Moniz, Raquel Duarte, Andreia Leite, Patrícia Soares, URBANTB group, Mário Carreira, Sofia Pereira, Catarina Alves, Filipe Alves, Ana Rodrigues, Ana Moreira, Márcia Cardoso, Sandra Mota, Ana Gomes, Liliana Ferreira, Marta Lopes, Isabel Correia, Juan Rachadell, Maria Gameiro, Ângela Dias, Manuel Pereira, Jorge Gonçalves, Maria Gonçalves, Adriana Taveira, Celene Neves, Lucinda Silva, Maria Mendes, Maria Teixeira, Maria Pereira, Milena Piedade, Antónia Teixeira, Carlos Carvalho

**Affiliations:** 1 NOVA National School of Public Health, Public Health Research Centre, NOVA University Lisbon, Lisbon, Portugal; 2 Central Lisbon Public Health Unit, Regional Health Administration of Lisbon and Tagus Valley, Lisbon, Portugal; 3 Multidisciplinary Unit for Biomedical Research, Biomedical Sciences Institute Abel Salazar, University of Porto, Oporto, Portugal; 4 NOVA National School of Public Health, Public Health Research Centre, Comprehensive Health Research Centre, NOVA University Lisbon, Lisbon, Portugal; 5 Epidemiological Investigation Unit, Public Health Institute, University of Porto, Oporto, Portugal; 6 Laboratory for Integrative and Translational Investigation in Populational Health, Oporto, Portugal; 7 Biomedical Sciences Institute Abel Salazar, University of Porto, Oporto, Portugal; 8 Clinical Investigation Unit, Regional Health Administration of the North, Oporto, Portugal; 9 Pneumology Service, Vila Nova de Gaia/Espinho Hospital Centre, Vila Nova de Gaia, Portugal; 10 Department of Epidemiology, National Institute of Health Doutor Ricardo Jorge, Lisbon, Portugal; 11 Centre for Vectors and Infectious Diseases Research, National Institute of Health Doutor Ricardo Jorge, Águas de Moura, Portugal

**Keywords:** Tuberculosis, Delayed diagnosis, Patient delay, Symptom perception, Symptom valorisation, Help-seeking behaviour

## Abstract

**Background:**

Diagnosis delay contributes to increased tuberculosis (TB) transmission and morbimortality. TB incidence has been decreasing in Portugal, but median patient delay (PD) has risen. Symptom valorisation may determine PD by influencing help-seeking behaviour. We aimed to analyse the association between symptom valorisation and PD, while characterising individuals who disregarded their symptoms.

**Methods:**

A cross-sectional study was conducted among TB patients in Lisbon and Oporto in 2019 – 2021. Subjects who delayed seeking care because they did not value their symptoms or thought these would go away on their own were considered to have disregarded their symptoms. PD was categorised using a 21-day cut-off, and a 30-day cut-off for sensitivity analysis. We estimated the effect of symptom valorisation on PD through a directed acyclic graph. Then, a multivariable regression analysis characterised patients that disregarded their symptoms, adjusting for relevant variables. We fitted Poisson regression models to estimate crude and adjusted prevalence ratios (PR).

**Results:**

The study included 75 patients. Median PD was 25 days (IQR 11.5–63.5), and 56.0% of participants had PD exceeding 21 days. Symptom disregard was reported by 38.7% of patients. Patients who did not value their symptoms had higher prevalence of PD exceeding 21 days compared to those who valued their symptoms [PR 1.59 (95% CI 1.05–2.42)]. The sensitivity analysis showed consistent point estimates but wider confidence intervals [PR 1.39 (95% CI 0.77–2.55)]. Being a smoker was a risk factor for symptom disregard [PR 2.35 (95% CI 1.14–4.82)], while living in Oporto [PR 0.35 (95% CI 0.16–0.75)] and having higher household incomes [PR 0.39 (95% CI 0.17–0.94)] were protective factors.

**Conclusions:**

These findings emphasise the importance of symptom valorisation in timely TB diagnosis. Patients who did not value their symptoms had longer PD, indicating a need for interventions to improve symptom recognition. Our findings also corroborate the importance of the socioeconomic determinants of health, highlighting tobacco as a risk factor both for TB and for PD.

**Supplementary Information:**

The online version contains supplementary material available at 10.1186/s12889-023-17319-7.

## Background

Tuberculosis (TB) remains one of the main causes of death worldwide [1]. In 2021, TB was the second cause of death regarding infectious diseases, only surpassed by COVID-19 [1]. In Portugal, the incidence rate of TB was, in 2021, 13.5 cases per 100,000 inhabitants [2]. Although it has been decreasing, it remains one of the highest in the European Union [1]. Diagnosis delay is an important contributor to disease transmission and is related to a higher morbimortality and the emergence of resistant mycobacteria [3–6]. Diagnosis delay corresponds to the period between symptom onset and diagnosis confirmation or treatment initiation. This period is typically divided in two components: patient delay (from symptom onset to the first medical appointment) and health services delay (from the first medical appointment to the diagnosis or treatment initiation) [7]. Shorter patient delays have been associated with male sex, younger age, higher education and higher knowledge about the disease [3, 7–11]. On the contrary, longer patient delays have been associated to being unemployed or homeless, having a lower income, residing in rural areas, and consuming tobacco, alcohol or other drugs [4, 12].

Unlike the incidence of the disease, the median patient delay has been rising in Portugal, from 40 days in 2010 to 51 days in 2021 [2]. The delay in seeking medical attention depends on the patient’s ability to recognise symptoms, acknowledgement of a possible illness, assessment of the need for professional care, and overcoming obstacles in obtaining that care [13]. Several interpretative theories have been developed to explain this process, proposing three phases in symptom appraisal: detection, interpretation and response [14]. Regarding detection and interpretation, previous studies assessing patient experiences demonstrated that when experiencing TB symptoms, patients frequently delayed their visit to the doctor because they considered symptoms like cough or lack of strength too unspecific or insufficient to motivate a medical appointment [3, 7, 10, 15]. Their perception was that they had a mild disease, likely a viral infection, that would resolve on its own [8–11]. In fact, the presence of mild or unspecific symptoms is associated with a longer patient delay and can be connected to lower symptom valorisation [16–18]. In previous studies, symptom valorisation has been reported as influenced by age, gender and sociocultural context [19–24]. Women frequently assume a role of managing their health and the health of their families, thus acquiring a higher degree of bodily awareness that influences symptom perception [19–21]. On the contrary, lower education was related to a lower capability of evaluating symptoms as a warning of a potentially serious disease [20]. This capability of recognising and interpreting symptoms also determines the individual’s attitudes when seeking healthcare, thus being critical for patient delay [13]. Additionally, the phase of responding to symptoms is importantly tied to the availability and access to healthcare, that is conditioned by economic and ethnic factors, as well as by stereotypes in relation to certain diseases, like human immunodeficiency virus (HIV), substance use disorders, cancer or TB [25].

Vast research on total, patient and health services delays have been conducted. However, few studies have assessed symptom valorisation and its effect on patient delay. Analysing this association while characterising the group of patients who disregard their symptoms is of utmost importance, since it provides useful information for developing public health interventions directed at shortening patient delay periods.

 With this study, we aim to analyse the association between symptom valorisation and patient delay (objective 1) and to characterise individuals that disregarded their symptoms (objective 2).

## Methods

### Study design and population

We conducted a cross-sectional study with an analytical component, considering all TB patients living in the metropolitan areas of Lisbon and Oporto, Portugal, as the target population.

### Recruitment and data collection

**Fig. 1:**
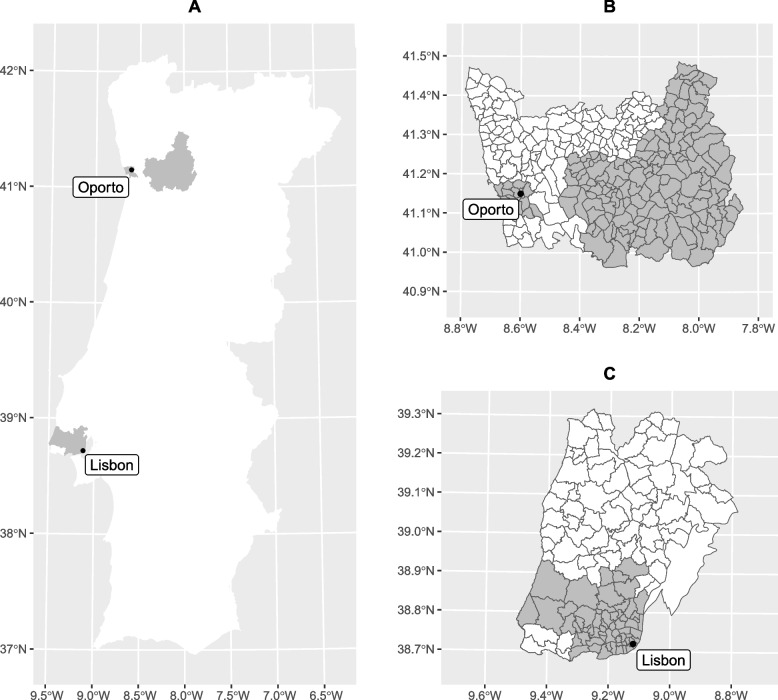
Healthcare clusters participating in the study. The shaded areas correspond to the zones covered by participating healthcare clusters (**A** – map of mainland Portugal; **B** – map of Oporto district [including Castelo de Paiva – Aveiro district, Celorico de Basto – Braga district, Cinfães and Resende – Viseu district]; **C** – map of Lisbon district)

Primary care facilities (healthcare clusters) from metropolitan areas of Lisbon and Oporto with the highest TB notification rates were invited to join the study [26]. Local public health units from the healthcare clusters accepting to participate (Fig. 1) were responsible for the recruitment and data collection process through the application of a questionnaire. All new cases of respiratory TB notified through the Portuguese epidemiological vigilance system (SINAVE), between August 2019 and August 2021, could be recruited. SINAVE is an electronic surveillance system operating at the national level in Portugal that allows professionals responsible for epidemiological vigilance to have instant access to notifications of mandatory declaration diseases. This accelerates the transmission of information and access to data [27].

The questionnaire employed was built based on a previously existent one developed by the World Health Organisation (WHO) [13] and aimed to identify the risk factors for a higher diagnosis delay of respiratory TB, both attributable to the patient or the health services. The questionnaire was divided in four sections regarding sociodemographic characterisation, description of the time corresponding to patient delay, description of the time corresponding to health services delay and characterisation of the level of knowledge about the disease and of the access to TB diagnostic and treatment centres.

### Eligibility criteria

For this analysis’ purpose, we excluded patients who were less than 18 years old, asymptomatic, or that were detected through contact tracing. We also excluded individuals with a patient delay below or equal to zero days, as well as those with a patient delay above 365 days as these were considered outliers. Patients with information missing on symptoms date, first medical appointment date or related to symptom disregard were also excluded.

### Variables

We selected the variables based on the literature review and the information from the questionnaire employed. The selected variables can be organised in dimensions: *sociodemographic* (age, gender, education, city of residence, household income, smoking habits and alcohol consumption frequency), *symptoms* (number of self-reported symptoms), *attitudes* (first initiative adopted by patients regarding their symptoms), *first medical appointment* (unit of the first medical appointment), and *knowledge about TB*. To evaluate the knowledge level about TB, we constructed a score that measured the number of correct answers to five questions regarding TB transmission, treatment, and prevention. These questions were based on an existing questionnaire developed by WHO [13]. There are differences regarding access to health services between regions in Portugal, therefore the city of residence can be used as a proxy to evaluate this aspect. The metropolitan area of Oporto has registered better indicators of access to healthcare compared to Lisbon, with a higher percentage of residents with an attributed family doctor (99% vs. 86%) [28]. The number of hospital appointments per inhabitant was also superior in Oporto (3.1 vs. 2.4) [29]. Additionally, the level of access differs by type of health service. Emergency services are more available than primary health care or TB treatment centres due to their constant accessibility, whereas these centres have limited hours. Detailed information about the included variables, possible values and respective questions from the questionnaire are available in Additional file 1.

#### Symptom valorisation and patient delay (objective 1)

To examine the association between symptom valorisation and patient delay, we defined the exposure variable (symptom valorisation) and the outcome variable (patient delay). Symptom valorisation was defined based on the patients’ answers to the question “*Which reasons do you consider to be associated with the time between the onset of symptoms and seeking medical help?*”. Those who responded “*I didn’t value the symptoms*” or “*I was convinced the symptoms would go away on their own*” were labelled as not having valued their symptoms. Patient delay was defined as the period, measured in days, between the symptom onset date and the first medical appointment date. This variable was dichotomised using a cut-off. The literature shows the most used cut-offs for patient delay are 21 and 30 days [5, 30]. For this analysis’ purpose, we assumed that there was a considerable diagnostic delay when the patient delay was superior to 21 days.

**Fig. 2:**
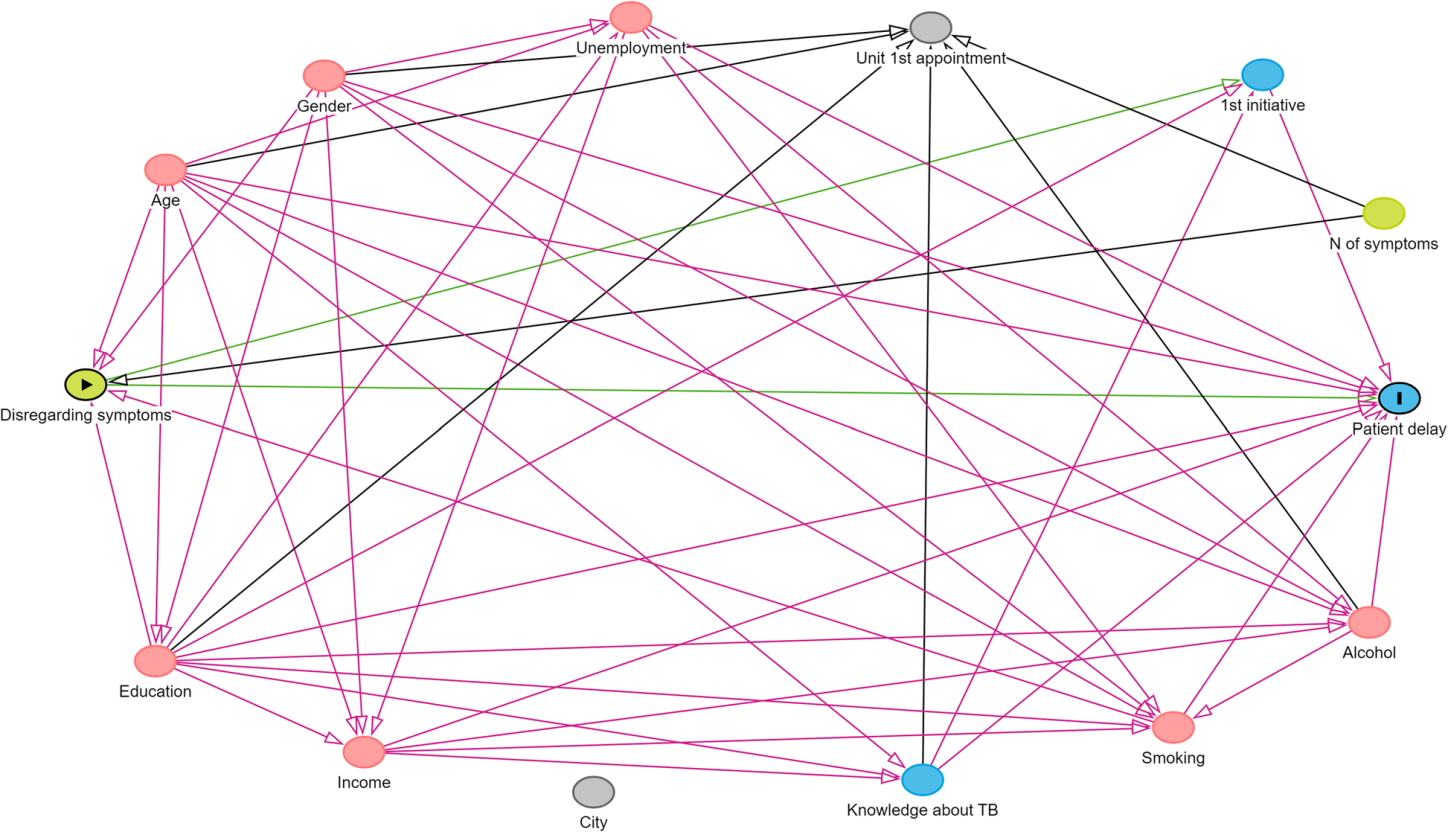
Representation of the DAG. The DAG identifies the minimal sufficient adjustment set necessary for estimating the effect of symptom valorisation on patient delay

We built a Directed Acyclic Graph (DAG) to identify the minimal sufficient adjustment set necessary for estimating the effect of symptom valorisation on patient delay, based on the “Evidence Synthesis for Constructing Directed Acyclic Graphs” (ESC-DAGs) methodology [31–33]. Briefly, the process encompasses two stages: mapping and translation. Mapping began with drawing a direct edge between the exposure (symptom valorisation) and the outcome (patient delay). The other study variables and all the possible connections between them and the exposure and the outcome were also represented, thus producing a saturated graph. Translation consisted of evaluating each connection represented by examining available literature selected after a search by keyword in Medline, Scopus and Google Scholar databases. A decision log was built to register the evidence supporting each connection and the respective direction (Additional file 2). The direct edges of the initial graph were retained, reversed or deleted accordingly, producing a DAG. The DAG was represented using *DAGgity* v3.0 software (Fig. 2). The minimal sufficient adjustment set obtained included age, gender, education, and smoking habits.

#### Characterisation of the individuals who disregarded their symptoms (objective 2)

Symptom valorisation variable was treated as the outcome to characterise individuals who disregarded their symptoms. The independent variables used for characterisation included the sociodemographic, symptoms, attitudes, first medical appointment, and knowledge about TB dimensions.

### Statistical analysis

#### Descriptive analysis

We described the sample using absolute and relative frequencies for categorical variables and measures of central tendency and dispersion for numeric variables. We also compared included and excluded individuals through hypothesis testing. For categorical variables, we used the Chi-square test, or Fisher’s exact test when more than 20% of the expected counts were less than 5. Numeric variables were assessed using the Student’s t-test or the Wilcoxon rank sum test in case the data followed or not a normal distribution, respectively. Observations with missing values were classified as “No Answer” (NA) and were not considered for the analysis.

#### Symptom valorisation and patient delay (objective 1)

According to previous studies, we anticipated a frequency of patient delay, defined as above 21 days, between 30 and 40% ( [30]. Thus, we fitted Poisson regression models with robust errors using sandwich estimation since these models do not have convergence problems and provide less biased estimates [34–37]. Crude and adjusted prevalence ratio (PR) were estimated with a 95% confidence interval (95% CI).

#### Characterisation of the individuals who disregarded their symptoms (objective 2)

Anticipating an elevated frequency of symptom disregard, we fitted Poisson regression models with robust errors using sandwich estimation. First, we performed crude analysis to evaluate the association between symptom valorisation and each variable selected for patient characterisation. This was followed by a multivariable analysis, adjusted by the variables that had a *p*-value inferior to 0.2 in the bivariable analysis. We also adjusted by other variables considered relevant to the analysis (e.g., age and gender). Crude and adjusted estimates of the PR with 95% CI were presented.

#### Sensitivity analysis

As above mentioned, there is not a consensual cut-off defining the ideal timing of TB diagnosis, with the most used cut-off points for the patient delay in literature being 21 and 30 days [5, 30]. Therefore, we also conducted a sensitivity analysis using the 30 days cut-off.

Statistical analysis was conducted with the support of R software version 4.2.2 [38].

## Results

**Fig. 3:**
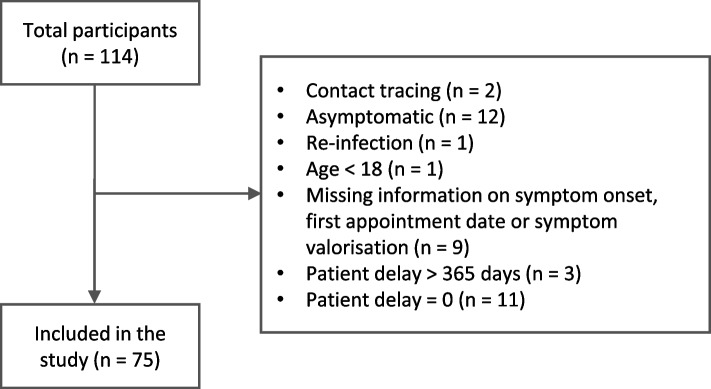
Process of exclusion of non-eligible individuals

The questionnaire was applied to 114 individuals, with 39 being excluded from the current study (Fig. 3).

This analysis included 75 patients with a median age of 50.0 (IQR 41.0 – 60.0). Most of the respondents were men (76.0%), resided in Oporto (58.7%), and the majority (91.7%) went to the doctor when first addressing their symptoms. We obtained a median patient delay of 25.0 days (IQR 11.5 – 63.5), with 56.0% of the participants having a patient delay superior to 21 days. Overall, the knowledge level about TB was good, with a median score of 4.0 out of 5.0 (IQR 3.0 – 5.0). Lack of symptom valorisation was verified in 38.7% of the patients (Table 1).

**Table 1:** Sociodemographic characteristics, knowledge about TB, attitude towards symptoms, and patient delay of the participants

**Variable**	***N* = 75**^a^
**Age**	
Median (IQR)^b^	50.0 (41.0, 60.0)
**Age categories**	
18—44	28 (37.3%)
45—64	36 (48.0%)
65 +	11 (14.7%)
**Gender**	
Men	57 (76.0%)
Women	18 (24.0%)
**City of residence**	
Lisbon	31 (41.3%)
Oporto	44 (58.7%)
**Education**	
4th grade	23 (31.1%)
9th grade	30 (40.5%)
Secondary/University	21 (28.4%)
Unknown^c^	1
**Unemployment**	
No	57 (78.1%)
Yes	16 (21.9%)
Unknown^c^	2
**Household income**	
650€ or less	15 (24.6%)
651—1000€	19 (31.1%)
More than 1000€	27 (44.3%)
Unknown^c^	14
**Smoking habits**	
No	29 (38.7%)
Ex-smoker	16 (21.3%)
Yes	30 (40.0%)
**Alcohol consumption frequency**	
Never	37 (49.3%)
Sometimes	9 (12.0%)
Regularly	29 (38.7%)
**First initiative addressing symptoms**	
Calling the emergency line	1 (1.4%)
Contacting a doctor outside the formal health system	1 (1.4%)
Going to the doctor	66 (91.7%)
Self-medicating	4 (5.6%)
Unknown^c^	3
**Unit of the first appointment**	
Emergency services	35 (46.7%)
Hospital	12 (16.0%)
Primary health care	28 (37.3%)
**Number of symptoms**	
Mean (SD)^b^	3.8 (1.5)
**Knowledge level about TB (0—5)**	
Median (IQR)^b^	4.0 (3.0, 5.0)
**Symptom valorisation**	
No	29 (38.7%)
Yes	46 (61.3%)
**Patient diagnosis delay**	
Median (IQR)^b^	25.0 (11.5, 63.5)
**Patient delay categorised on 21 days**	
Delayed	42 (56.0%)
Not delayed	33 (44.0%)
**Patient delay categorised on 30 days**	
Delayed	32 (42.7%)
Not delayed	43 (57.3%)

^a^n (%)

^b^*IQR* Interquartile range, *SD* Standard deviation

^c^Unknown values were not considered in the calculus of the percentages

The included and the excluded groups differed in the number of symptoms reported (*p* < 0.001), first appointment unit (*p* = 0.002), symptom valorisation (*p* < 0.001) and patient delay categorised on 21 days (*p* = 0.006). The remaining variables had no significant differences (Additional file 3).

### Symptom valorisation and patient delay (objective 1)

Lack of symptoms valorisation was significantly associated with a patient delay superior to 21 days (Table 2). Patients who did not value their symptoms had 1.59 times the prevalence of patient delay above 21 days compared to patients who did value their symptoms [PR 1.59 (95% CI 1.05 – 2.42)]. On the contrary, the sensitivity analysis, which considered patient delay using the 30 days cut-off, showed consistent point estimates but with wider confidence intervals (Table 2).

**Table 2:** Association between symptom valorisation and patient delay in urban Portugal (crude and adjusted prevalence ratios with 95% confidence intervals)

	**Crude analysis (*n* = 75)**			**Adjusted analysis**^**b**^** (*n* = 74)**		
	**PR**^a^	**95% CI**^a^	***p*-value**	**PR**^a^	**95% CI**^a^	***p*-value**
Patient delay with a 21-day cut-off						
**Symptom valorisation** (Ref. Yes)	1.44	0.98, 2.13	0.066	1.59	1.05, 2.42	**0.029**
Patient delay with a 30-day cut-off						
**Symptom valorisation** (Ref. Yes)	1.23	0.73, 2.09	0.433	1.39	0.77, 2.51	0.268

^a^*PR* Prevalence Ratio, *CI* Confidence Interval

^b^Adjusted by age, gender, education, and smoking habits

### Characterisation of the individuals who disregarded their symptoms (objective 2)

We found a significant association between symptom valorisation and city of residence, monthly household income, and smoking habits (Table 3). The individuals who smoked had a prevalence of symptom disregard 1.35 times bigger than non-smokers [PR 2.35 (95% CI 1.14 – 4.82)]. On the other hand, patients residing in Oporto had a prevalence of lack of symptoms valorisation 0.35 times the prevalence of those residing in Lisbon [PR 0.35 (95% CI 0.16 – 0.75)]. For those with a household income superior to 1000€ per month, the prevalence of lack of symptoms valorisation was 0.39 times the prevalence of those earning 650€ or less [PR 0.39 (95% CI 0.17 – 0.94)]. No other characteristic was demonstrated to significantly describe these patients.

**Table 3:** Characteristics associated with disregarding TB symptoms in urban Portugal (crude and adjusted prevalence ratios with 95% confidence intervals)

**Characteristic**	**Crude analysis**			**Adjusted analysis**^**b**^		
	**PR**^a^	**95% CI**^a^	***p*-value**	**PR**^a^	**95% CI**^a^	***p*-value**
**Age categories** (Ref. 18—44)						
45—64	0.78	0.41, 1.47	0.437	0.54	0.25, 1.16	0.113
65 +	1.06	0.49, 2.32	0.883	0.75	0.38, 1.49	0.411
**Gender** (Ref. Men)						
Women	1.42	0.79, 2.56	0.236	1.32	0.47, 3.74	0.596
**City of residence** (Ref. Lisbon)						
Oporto	0.50	0.28, 0.89	**0.019**	0.35	0.16, 0.75	**0.007**
**Education** (Ref. 4^th^ grade)						
9^th^ grade	1.02	0.52, 2.01	0.949			
Secondary/University	0.97	0.46, 2.06	0.944			
**Unemployment** (Ref. No)						
Yes	1.36	0.74, 2.47	0.319			
**Household income** (Ref. 650€ or less)						
651–1000€	0.99	0.52, 1.88	0.968	1.93	0.87, 4.30	0.108
More than 1000€	0.35	0.14, 0.88	**0.026**	0.39	0.16, 0.94	**0.037**
**Smoking habits** (Ref. No)						
Ex-smoker	0.60	0.19, 1.93	0.396	0.69	0.17, 2.76	0.600
Yes	1.83	0.97, 3.43	0.061	2.35	1.14, 4.82	**0.020**
**Alcohol consumption frequency** (Ref. Never)						
Sometimes	2.06	1.06, 3.98	**0.032**	3.21	0.72, 14.2	0.125
Regularly	1.17	0.60, 2.27	0.643	1.54	0.58, 4.05	0.382
**Unit of the first appointment** (Ref. Emergency services)						
Hospital	0.39	0.10, 1.47	0.164	1.00	0.47, 2.15	0.991
Primary health care	1.00	0.56, 1.78	> 0.999	1.35	0.72, 2.56	0.353
**Number of symptoms**	1.08	0.90, 1.30	0.391			
**Knowledge level about TB (0—5)**	1.12	0.86, 1.46	0.407			

^a^*PR* Prevalence Ratio, *CI* Confidence Interval

^b^(*N* = 61). Adjusted by age, gender, city of residence, household income, smoking habits, alcohol consumption frequency, and unit of the first appointment

## Discussion

Our findings reveal that patients who did not value their symptoms had a significantly higher proportion of patient delay superior to 21 days than patients who valued their symptoms. Additionally, we identified that smokers had a higher prevalence of not valuing their symptoms while living in Oporto and higher household incomes were associated with symptom valorisation.

The association between symptom valorisation and patient delay highlights the importance of symptom recognition in the timely diagnosis of TB. The patients who did not value their symptoms may have perceived them as mild and the severity of their disease to be low, as has been previously reported in the literature [10, 11]. TB initial symptoms are often interpreted as normal or common cold, which, allied to self-medicating, leads to patient diagnosis delay [3, 39]. These patients may not have considered themselves at risk of developing TB, therefore not seeking prompt medical care [17]. Hence, measures for increasing public awareness about the symptoms of TB and emphasising the need to seek early care should be developed and implemented.

Regarding the cut-offs used to categorise patient delay, there is no established period of diagnosis delay that is deemed to be acceptable. However, from a disease transmission control point of view, the period for total diagnosis delay should not surpass four weeks (28 days) [12], hence the period for patient delay should be inferior. In this case, it is likely that the 30-day cut-off was too wide, classifying prolonged periods as acceptable. The 21-day cut-off is perhaps more accurate at identifying prolonged patient delays, hence a reference cut-off for patient delay should not be superior to 21 days. Nevertheless, we also note that the obtained confidence intervals were quite wide, so it is possible that this study might not have had sufficient power. Further investigation in defining cut-offs for diagnosis delay is needed, as it is useful both for academic research and clinical practice [5]. Also, if patients were provided with an accurate cut-off from which they knew they should seek medical care regarding their symptoms, the patient delay period could decrease, assuming that timely access to healthcare is guaranteed [5].

Although symptom disregard has been related to a possible lack of knowledge about the disease [40] or a lower level of education [20], neither were significant in our analysis. Nevertheless, we identified other sociodemographic factors associated with symptom valorisation. Patients who lived in Oporto valued symptoms more than those living in Lisbon. Marco de Canaveses and Penafiel, two cities in Oporto metropolitan area, present the highest TB incidence rates in the country (respectively, 52.7 and 55.9 cases per 100,000 inhabitants) [2]. Hence Oporto population may be more aware of the existence of this disease, valuing its symptoms [2]. Besides, the Oporto metropolitan area has registered better indicators of access to healthcare when compared to Lisbon [28]. In 2019, in Portugal, the North region had the highest percentage of patients with an assigned family doctor (98.4%). On the contrary, Lisbon and Tagus Valley had the lowest (85.6%) [28]. Despite this difference, we do not think this may have affected our results, given that none of our participants referred difficulties scheduling a medical appointment, neither because there was a delay of the services nor because these services were not available or distant. Nevertheless, in 2021, 52.7% of the TB patients in the Lisbon district were immigrants, as opposed to only 5.8% in the Oporto district [2]. The immigrant population represents a challenge to implementing TB control programs, as it is a vulnerable group with inherent difficulties accessing healthcare [41]. Immigrant population showed, in 2021, a TB notification rate 3.8 times higher than the national average (55.8 per 100,000 inhabitants), with a progressive increase in the proportion of cases, reaching 25.8% in the same year [2]. Therefore, the regional asymmetries regarding symptom valorisation should be addressed in future investigations to clarify whether these are linked to unequal healthcare access or other non-explored factors. Patients who earned a higher household income also had a lower prevalence of lack of symptom valorisation. Earning a higher household income has been associated with higher education and higher health literacy levels [42, 43], which promotes appropriate help-seeking behaviour and improves access to healthcare [44–46]. On the opposite, patients who smoked had a higher proportion of symptom disregard. Although being more likely to experience respiratory symptoms than non-smokers, smokers are less concerned by these, therefore they do not seek the help they require [47]. This is also true for other chronic diseases that, like pulmonary TB, are characterised by persistent cough [7]. These findings suggest the need for targeted health education interventions to improve symptom recognition and valorisation, especially among patients with chronic respiratory diseases or smokers. This is important in the studied regions, with the metropolitan area of Lisbon presenting, in 2019, a prevalence of smokers of 18.2% and the North region, a prevalence of 16.2% [48].

Our study has some limitations. We could not establish temporal relationships due to its design. Additionally, we were working with information that was self-reported by the patients, which may have introduced a recall bias. For example, patients who did not value their symptoms may be less precise in reporting their onset of symptoms date. In fact, this date is hard to define, as it is, regarding TB’s frequent insidious presentation, which may underestimate patient delay. Moreover, the considered outliers of patient delay of zero or superior to 365 days are, in fact, possible. Even so, we considered that, especially for typically insidious diseases like TB, the first phases of symptom appraisal (detection and interpretation that lead to a response) should take more than 24 h, hence classifying zero days patient delays as unplausible. Likewise, we viewed as unlikely that patient delays superior to 365 days should occur and that these could represent errors in the introduction of the symptom onset or first medical appointment dates, either during data collection or during its introduction in the database, that could have tampered with our results. Also, the process of recruitment of participants was below expectations. In 2020 only, in Lisbon and Oporto districts, there was a total of 809 new TB notifications [26]. During the whole data collection period, we were only able to assemble 114 respondents. The data collection occurred during the COVID-19 pandemic when our interviewers from local public health units were deviated to other tasks related to pandemic control, thus conditioning the application of the questionnaire. In fact, we only registered five answered questionnaires during the pandemic period, therefore we consider that the impact of this context in our results was mainly in terms of a lower number of participants and was not related to an overestimation of patient delay, for example, due to the conditioned response of healthcare services during the pandemic. Despite the low participation, our sample has similar characteristics when compared to the population of TB patients in the studied regions: approximately 70% of the cases are men, with a median age of 50 years old [2, 4]. Moreover, the existence of missing information in some variables lead to a smaller sample in the multivariable analysis. Finally, we did not find an association between symptom valorisation and the unit of the first appointment. However, the interpretation of the answer options to the question that originated this variable is not unequivocal. The option “hospital” was meant for a non-urgent medical appointment at a hospital setting, though we cannot assure that this was explained during data collection and that subjects responded accordingly. There may have been a misclassification where people intended to answer “hospital emergency services” but instead answered “hospital” which prevents us from detecting an association.

This study also has several strengths. To the best of our knowledge, it is the first study to directly evaluate symptom valorisation and its association with TB patient delay in Portugal, providing new and valuable information. We also identified some factors associated with symptom valorisation, offering a base for future targeted health education campaigns to reduce patient delay, particularly among vulnerable populations.

## Conclusions

TB remains a significant global public health issue and timely diagnosis is crucial for reducing its transmission, morbidity and mortality. We found that a lack of symptom valorisation was associated with longer patient delay periods and identified that living in Oporto and higher household incomes were associated with symptom valorisation while smokers had a higher prevalence of symptom disregard. These findings emphasise the importance of the socioeconomic determinants of health and draw attention to tobacco as a risk factor both for TB and for diagnosis delay, justifying the implementation of anti-tobacco measures and interventions. Additionally, targeted education campaigns to improve symptom recognition should be implemented, mainly regarding smokers or patients with respiratory chronic diseases. It should be explained to patients that a new, persistent cough or a change in their usual cough pattern should alert them to seek medical care, as it may represent a potentially serious illness (flu, COVID-19, cancer or TB, for example).

### Supplementary Information

**Additional file 1: Supplementary Table 1.** Variable operationalisation. Detailed variables, possible values, respective questions from the questionnaire and other practical aspects.

**Additional file 2: Supplementary Table 2. **Directed Acyclic Graph. Decision log used for the construction of the directed acyclic graph.

**Additional file 3: Supplementary Table 3.** Comparing included and excluded subjects. Comparative analysis with hypothesis tests between individuals included and excluded of the study.

## Data Availability

The data that support the findings of this study are available from NOVA National School of Public Health, but restrictions apply to the availability of these data, which were used under license for the current study, and so are not publicly available. Data are however available from the authors upon reasonable request and with permission of NOVA National School of Public Health. To request the data, please contact the first author, Margarida de Morais, at margarida.m.morais@gmail.com.

## Abbreviations

CI: Confidence interval
COVID – 19: Coronavirus disease 2019
DAG: Directed acyclic graph
ESC-DAGs: Evidence Synthesis for Constructing Directed Acyclic Graphs
HIV: Human immunodeficiency virus
IQR: Interquartile range
NA: No answer
PD: Patient delay
PR: Prevalence ratio
SD: Standard deviation
SINAVE: Sistema Nacional de Vigilância Epidemiológica (National System of Epidemiological Vigilance)
TB: Tuberculosis
WHO: World Health Organisation
