# Supplementary material for: Investigating the role of symptom valorisation in tuberculosis patient delay in urban areas in Portugal
Source: BMC Public Health. 2023 Dec 5;23:2421. doi: 10.1186/s12889-023-17319-7 (PMC10696854; doi:10.1186/s12889-023-17319-7)
Supplement: Supplementary file 1 — Additional file 1: Supplementary Table 1. Variable operationalisation. Detailed variables, possible values, respective questions from the questionnaire and other practical aspects. [file 12889_2023_17319_MOESM1_ESM.docx]

# Variable operationalisation

**Supplementary table 1 – Variable operationalisation.** Detailed variables, possible values, respective questions from the questionnaire and other practical aspects

| Variable | Values | Question | Recoding/Practical aspects |
| --- | --- | --- | --- |
| Age | 18-120 | 8. Birth date: |  |
| Age categories | - 18-44 - 45-64 - 65+ | 8. Birth date: |  |
| Gender | - Men - Women | 9. Gender:   - Masculine - Feminine - Other | - No one answered “Other”. |
| Education | - 4^th^ grade - 9^th^ grade - Secondary/University | 11. What was the highest education level that you have completed?   - Cannot read nor write - Can read but have not completed the 4^th^ grade - 4^th^ grade - 6^th^ grade - 9^th^ grade - 12^th^ grade - Bachelor or licentiate degree - Masters or doctorate degree | - No one answered the first two options. - 6^th^ and 9^th^ grades were grouped into “9^th^ grade”. - 12^th^ grade, bachelor, licentiate, masters or doctorate degrees were grouped into “Secondary/University”. |
| City of residence | - Lisbon - Oporto | - 16. Parish of residence: | - Parishes were grouped by city. |
| Household income | - 650€ or less - 651-1000€ - More than 1000€ | 13. What is your monthly household income (including rents or pensions)?   - <650€ - 651-1000€ - 1001-1500€ - 1501-2000€ - 2001-2500€ - >2500€ | - 1001-1500€, 1501-2000€, 2001-2500€, <2500€ were recoded as “More than 1000€”. |
| Smoking habits | - No - Ex-smoker^[[1]](#endnote-2)^ - Yes | 19. Do you smoke or have smoked in the past?   - Yes - No   19.1. In the last 3 months, how often did you smoke?   - Never   - How long have you quit? - Occasionally - Daily | - Responding “No” to Q.19 was coded as “No”. - Responding “Never” to Q.19.1 was coded as “Ex-smoker”^.^ - Responding “Occasionally” or “Daily” to Q19.1 was coded as “Yes”. |
| Alcohol consumption frequency | - Never - Sometimes - Regularly | 20.1. In the last 3 months, how often have you consumed alcoholic beverages?   - Never - Once a month or less - 2 to 4 times per month - 2 to 3 times per week - 4 or more times per week | - Responding “Never” was coded as “Never”. - “Sometimes” corresponds to consuming alcoholic beverages once a month or less or 2 to 4 times per month. - “Regularly” corresponds to consuming alcoholic beverages 2 to 3 times per week or 4 or more times per week. |
| First initiative addressing symptoms | - Calling the emergency line - Contacting a doctor outside the formal health system - Going to the doctor - Self-medicating | 26. What was your first initiative seeking help for your current health condition?   - Going to the doctor - Contacting a doctor outside of the formal health system (e.g., a friend) - Contacting a nurse - Contacting a pharmacist - Calling the emergency line - Alternative therapies - Self-medicating | - There were no answers as “Contacting a nurse”, “Contacting a pharmacist” and “Alternative therapies”. |
| Unit of the first appointment | - Emergency services - Hospital - Primary health care | 33. What was the health unit of your first medical appointment?   - Primary health care - Hospital emergency service - Public - Private - Hospital   - Public   - Private - Private clinic - Tuberculosis diagnosis centre | - The distinction between private and public services was not made. - “Hospital emergency service” was recoded as “Emergency services”. - “Private clinic” was recoded as “Primary health care”. - “Tuberculosis diagnosis centre” was recoded as “Hospital”. |
| Number of self-reported symptoms | 0-15 | 22. Which symptoms do you remember having had?   - Asymptomatic - Dry cough - Cough with sputum - Cough with blood - Persistent fever (3 or more days) - Weight loss - Thoracic pain - Lack of strength - Night sweats | - Sum of all reported symptoms. |
| Knowledge level about tuberculosis | 0-5 | 45.2. Is TB a hereditary disease?  45.3. Is TB contagious?  45.4. Is TB curable?  45.5 Is there a vaccine for TB?  Answer options (45.2-45.5):   - Yes - No - Does not know - Does not want to answer   45.6 What is the approximate duration of the treatment of TB (non-multi-resistant)?  (open answer) | Correct answers considered:  45.2. No.  45.3. Yes.  45.4. Yes.  45.5. Yes.  45.6. All answers between 6 and 12 months.  A score that measured the number of correct answers given to these five questions was created. Each question had equal weight to the score, that could range from 0 (no correct answers) to 5 (all answers were correct). “Does not know” and “Does not want to answer” were considered wrong answers. |
| Symptom valorisation | - Yes - No | 34. Which reasons do you consider to be associated with the time between the onset of symptoms and seeking medical help? (Choose one)   - Non applicable (the delay was zero) - Advice given by the first entity approached (emergency line, doctor) - Delay in scheduling the medical appointment - Fear of the diagnosis/going to the doctor - Conviction that the symptoms would go away on their own - Did not value the symptoms - Fear of stigma - Shame - Low confidence in health services - Health services not available or distant - Difficulty in attending the appointment   - I depended on someone else   - I had people at my guard that needed my care   - Lack of transportation means   - I was not granted work leave   - Lack of time for professional reasons - Economic limitations - Reclusion situation - Does not speak Portuguese | - “Conviction that the symptoms would go away on their own” and “Did not value the symptoms” were coded as “No”. - All other options were coded as “Yes”. |
| Patient delay | 1-365 | 23. What was the date of the first symptom?  31. What was the date of the first medical appointment? | Period in days since onset of symptoms to the date of the first medical appointment. |
| Patient delay in relation to the 21 days cut-off | - Not delayed - Delayed | 23. What was the date of the first symptom?  31. What was the date of the first medical appointment? | - “Not delayed” corresponds to a patient delay of 21 or less days. - “Delayed” means patient delay was superior to 21 days. |

1. We considered ex-smokers individuals who had not smoked in the past 3 months or longer. [↑](#endnote-ref-2)
