# Supplementary material for: Investigating the role of symptom valorisation in tuberculosis patient delay in urban areas in Portugal
Source: BMC Public Health. 2023 Dec 5;23:2421. doi: 10.1186/s12889-023-17319-7 (PMC10696854; doi:10.1186/s12889-023-17319-7)
Supplement: Supplementary file 2 — Additional file 2: Supplementary Table 2. Directed Acyclic Graph. Decision log used for the construction of the directed acyclic graph. [file 12889_2023_17319_MOESM2_ESM.docx]

# Directed Acyclic Graph – Decision Log

**Supplementary table 2 – Directed Acyclic Graph.** Decision log used for the construction of the directed acyclic graph

| Node 1 | Node 2 | Direction | Justification | Reference |
| --- | --- | --- | --- | --- |
| Disregarding symptoms | Patient delay | 1 to 2 | EXPOSURE TO OUTCOME | - |
| Disregarding symptoms | Age | 2 to 1 | Symptom perception is influenced by age, gender, and sociocultural context. | (1-3) |
| Disregarding symptoms | Gender | 2 to 1 | Women in Western societies are given the role of surveilling their health and the health of their families, which confers them a higher degree of bodily awareness and influences symptom perception. | (1, 3, 4) |
| Disregarding symptoms | Education | 2 to 1 | People with lower education consistently had a lower capability of evaluating symptoms as a warning of a potentially serious disease. | (2) |
| Disregarding symptoms | Income | No connection | - | - |
| Disregarding symptoms | Unemployment | No connection | - | - |
| Disregarding symptoms | Alcohol consumption | No connection | - | - |
| Disregarding symptoms | Smoking habits | 2 to 1 | Although being more likely to experience respiratory symptoms than non-smokers, smokers are less concerned by these symptoms, therefore not seeking the required help. | (5) |
| Disregarding symptoms | Number of symptoms | 2 to 1 | The more symptoms an individual has, the more likely it is for him to value them. | (6, 7) |
| Disregarding symptoms | City of residence | No connection | - | - |
| Disregarding symptoms | 1^st^ initiative | 1 to 2 | The perceived severity of the disease was associated with the type of care chosen by the patient. | (8, 9) |
| Disregarding symptoms | Knowledge about tuberculosis | No connection | - | - |
| Disregarding symptoms | Unit of the first appointment | No connection | - | - |
| Patient delay | Age | 2 to 1 | Younger age is associated with lower patient delay. | (10-12) |
| Patient delay | Gender | 2 to 1 | Female gender’s likelihood of extreme patient delay was higher when compared to men. | (13) |
| Patient delay | Education | 2 to 1 | Uneducated patients had higher odds of patient delay. | (14) |
| Patient delay | Income | 2 to 1 | Low income was associated with patient delay in tuberculosis diagnosis. | (11, 15) |
| Patient delay | Unemployment | 2 to 1 | Being unemployed was identified as a risk factor for patient delay in TB diagnosis. | (11, 12, 16-19) |
| Patient delay | Alcohol consumption | 2 to 1 | Alcohol and tobacco consumption are associated with higher patient delays. | (12, 16, 20) |
| Patient delay | Smoking habits | 2 to 1 | Alcohol and tobacco consumption are associated with higher patient delays. | (12, 16, 20) |
| Patient delay | Number of symptoms | No connection | - | - |
| Patient delay | City of residence | No connection | - | - |
| Patient delay | 1^st^ initiative | 2 to 1 | Self-medication, as well as seeking initial care from informal providers, were associated with longer patient delays. | (11, 14, 21-23) |
| Patient delay | Knowledge about tuberculosis | 2 to 1 | Misconceptions about TB transmission were associated with patient delay in diagnosis and poor treatment outcomes. | (6, 10, 11, 15, 22, 23) |
| Patient delay | Unit of the first appointment | No connection | - | - |
| Age | Gender | No connection | - | - |
| Age | Education | 1 to 2 | Younger individuals have completed a higher level of education as opposed to those older than 65. | (24) |
| Age | Income | 1 to 2 | Medium remuneration is lower for individuals aged 18 to 24. | (25) |
| Age | Unemployment | 1 to 2 | Unemployment rates have been superior for individuals who are 25 years old or younger than in other age groups, in Portugal, in the last 20 years. | (26) |
| Age | Alcohol consumption | 1 to 2 | Age, gender, and socio-economic status were identified as risk factors for alcohol consumption. | (27) |
| Age | Smoking habits | 1 to 2 | Tobacco smoking was significantly associated with age. | (28, 29) |
| Age | Number of symptoms | No connection | - | - |
| Age | City of residence | No connection | - | - |
| Age | 1^st^ initiative | No connection | - | - |
| Age | Knowledge about tuberculosis | 1 to 2 | Knowledge of TB was higher for the age category 45–49 years old as opposed to the youngest age group (15–24 years). | (30, 31) |
| Age | Unit of the first appointment | 1 to 2 | People diagnosed with tuberculosis in emergency facilities were younger than people diagnosed in primary care or hospital appointments. Also, they were mostly males, had low education and alcohol abuse. | (32) |
| Gender | Education | 1 to 2 | Approximately 61% of the individuals with a superior education in Portugal are women. However, most people with no education at all are also women (71%). This contradiction can be explained by age differences, with younger women being more educated. | (33) |
| Gender | Income | 1 to 2 | Remuneration of men was higher than that of women in Portugal in the last 20 years. | (34) |
| Gender | Unemployment | 1 to 2 | The unemployment rate among women was higher than that of men in the last 20 years in Portugal. | (35) |
| Gender | Alcohol consumption | 1 to 2 | Age, gender, and socio-economic status were identified as risk factors for alcohol consumption. | (27) |
| Gender | Smoking habits | 1 to 2 | There is higher smoking behaviour among males than females; males were more likely to smoke more and more regularly than females. | (36, 37) |
| Gender | Number of symptoms | No connection | - | - |
| Gender | City of residence | No connection | - | - |
| Gender | 1^st^ initiative | No connection | - | - |
| Gender | Knowledge about tuberculosis | No connection | - | - |
| Gender | Unit of the first appointment | 1 to 2 | People diagnosed with tuberculosis in emergency facilities were younger than people diagnosed in primary care or hospital appointments. Also, they were mostly males, had low education and alcohol abuse. | (32) |
| Education | Income | 1 to 2 | Remuneration is higher among workers with higher qualifications. | (38-40) |
| Education | Unemployment | 1 to 2 | Individuals with lower education present higher unemployment levels. | (41) |
| Education | Alcohol consumption | 1 to 2 | Moderate and heavy drinkers were more frequently men, older and less educated. | (42) |
| Education | Smoking habits | 1 to 2 | Education level was significantly associated with tobacco consumption. | (28, 29) |
| Education | Number of symptoms | No connection | - | - |
| Education | City of residence | No connection | - | - |
| Education | 1^st^ initiative | 1 to 2 | A higher education level was associated with appropriate health-seeking behaviour. | (43, 44) |
| Education | Knowledge about tuberculosis | 1 to 2 | A low score regarding TB knowledge was associated with age and having less than 12 years of education. | (45, 46) |
| Education | Unit of the first appointment | 1 to 2 | People diagnosed with tuberculosis in emergency facilities were younger than people diagnosed in primary care or hospital appointments. Also, they were mostly males, had low education and alcohol abuse. | (32) |
| Income | Unemployment | 2 to 1 | The family income of the unemployed is lower than the family income of their salary earning peers. | (47) |
| Income | Alcohol consumption | 1 to 2 | Income was associated independently with alcohol use and consume frequency. | (48, 49) |
| Income | Smoking habits | 1 to 2 | The probability of smoking for both males and females is significantly associated with income. | (29) |
| Income | Number of symptoms | No connection | - | - |
| Income | City of residence | No connection | - | - |
| Income | 1^st^ initiative | No connection | - | - |
| Income | Knowledge about tuberculosis | 1 to 2 | Having better knowledge about TB was associated with income. | (31, 50) |
| Income | Unit of the first appointment | No connection | - | - |
| Unemployment | Alcohol consumption | 1 to 2 | Unemployment was demonstrated to have a positive effect on drinking behaviour. | (51) |
| Unemployment | Smoking habits | 1 to 2 | There was a higher rate of tobacco use among the unemployed. | (52) |
| Unemployment | Number of symptoms | No connection | - | - |
| Unemployment | City of residence | No connection | - | - |
| Unemployment | 1^st^ initiative | No connection | **-** | - |
| Unemployment | Knowledge about tuberculosis | No connection | - | - |
| Unemployment | Unit of the first appointment | No connection | - | - |
| Alcohol consumption | Smoking habits | 1 to 2 | Alcohol consumption, through its disinhibiting effect, leads to an increase in tobacco use. Drinkers have a higher probability of smoking regularly. | (28, 53, 54) |
| Alcohol consumption | Number of symptoms | No connection | - | - |
| Alcohol consumption | City of residence | No connection | - | - |
| Alcohol consumption | 1^st^ initiative | No connection | - | - |
| Alcohol consumption | Knowledge about tuberculosis | No connection | - | - |
| Alcohol consumption | Unit of the first appointment | 1 to 2 | People diagnosed with tuberculosis in emergency facilities were younger than people diagnosed in primary care or hospital appointments. Also, they were mostly males, had low education and alcohol abuse. | (32) |
| Smoking habits | Number of symptoms | No connection | - | - |
| Smoking habits | City of residence | No connection | - | - |
| Smoking habits | 1^st^ initiative | No connection | - | - |
| Smoking habits | Knowledge about tuberculosis | No connection | - | - |
| Smoking habits | Unit of the first appointment | No connection | - | - |
| Number of symptoms | City of residence | No connection | - | - |
| Number of symptoms | 1^st^ initiative | No connection | - | - |
| Number of symptoms | Knowledge about tuberculosis | No connection | - | - |
| Number of symptoms | Unit of the first appointment | 1 to 2 | Seeking hospital level care was associated with having more tuberculosis symptoms. | (55) |
| City of residence | 1^st^ initiative | No connection | - | - |
| City of residence | Knowledge about tuberculosis | No connection | - | - |
| City of residence | Unit of the first appointment | No connection | - | - |
| 1^st^ initiative | Knowledge about tuberculosis | 2 to 1 | Higher knowledge about TB was associated with seeking care at health facilities with proper diagnostic capacity. | (9, 56) |
| 1^st^ initiative | Unit of the first appointment | No connection | - | - |
| Knowledge about tuberculosis | Unit of the first appointment | 1 to 2 | Having a higher knowledge about TB was associated with seeking hospital care. | (9, 55) |

**References**

1. Malterud K, Guassora AD, Graungaard AH, Reventlow S. Understanding medical symptoms: a conceptual review and analysis. Theor Med Bioeth. 2015;36(6):411-24.

2. Sarma EA, Rendle KA, Kobrin SC. Cancer symptom awareness in the US: Sociodemographic differences in a population-based survey of adults. Prev Med. 2020;132:106005.

3. Whitaker KL, Scott SE, Wardle J. Applying symptom appraisal models to understand sociodemographic differences in responses to possible cancer symptoms: a research agenda. Br J Cancer. 2015;112 Suppl 1(Suppl 1):S27-34.

4. Rosendal M, Jarbøl DE, Pedersen AF, Andersen RS. Multiple perspectives on symptom interpretation in primary care research. BMC Fam Pract. 2013;14:167.

5. Walabyeki J, Adamson J, Buckley HL, Sinclair H, Atkin K, Graham H, et al. Experience of, awareness of and help-seeking for potential cancer symptoms in smokers and non-smokers: A cross-sectional study. PLoS One. 2017;12(8):e0183647.

6. Almeida CP, Skupien EC, Silva DR. Health care seeking behavior and patient delay in tuberculosis diagnosis. Cad Saude Publica. 2015;31(2):321-30.

7. Elliott AM, McAteer A, Hannaford PC. Revisiting the symptom iceberg in today's primary care: results from a UK population survey. BMC Fam Pract. 2011;12:16.

8. Begashaw B, Tessema F, Gesesew HA. Health Care Seeking Behavior in Southwest Ethiopia. PLoS One. 2016;11(9):e0161014.

9. Engeda EH, Dachew BA, Kassa Woreta H, Mekonnen Kelkay M, Ashenafie TD. Health Seeking Behaviour and Associated Factors among Pulmonary Tuberculosis Suspects in Lay Armachiho District, Northwest Ethiopia: A Community-Based Study. Tuberc Res Treat. 2016;2016:7892701.

10. Alene M, Assemie MA, Yismaw L, Gedif G, Ketema DB, Gietaneh W, et al. Patient delay in the diagnosis of tuberculosis in Ethiopia: a systematic review and meta-analysis. BMC Infect Dis. 2020;20(1):797.

11. Eltayeb D, Pietersen E, Engel M, Abdullahi L. Factors associated with tuberculosis diagnosis and treatment delays in Middle East and North Africa: a systematic review. East Mediterr Health J. 2020;26(4):477-86.

12. Helfinstein S, Engl E, Thomas BE, Natarajan G, Prakash P, Jain M, et al. Understanding why at-risk population segments do not seek care for tuberculosis: a precision public health approach in South India. BMJ Glob Health. 2020;5(9).

13. Díez M, Bleda MJ, Alcaide J, Caloto T, Castells C, Cardenal JI, et al. Determinants of patient delay among tuberculosis cases in Spain. Eur J Public Health. 2004;14(2):151-5.

14. Getnet F, Demissie M, Assefa N, Mengistie B, Worku A. Delay in diagnosis of pulmonary tuberculosis in low-and middle-income settings: systematic review and meta-analysis. BMC Pulm Med. 2017;17(1):202.

15. Fuge TG, Bawore SG, Solomon DW, Hegana TY. Patient delay in seeking tuberculosis diagnosis and associated factors in Hadiya Zone, Southern Ethiopia. BMC Res Notes. 2018;11(1):115.

16. Santos JA, Leite A, Soares P, Duarte R, Nunes C. Delayed diagnosis of active pulmonary tuberculosis - potential risk factors for patient and healthcare delays in Portugal. BMC Public Health. 2021;21(1):2178.

17. Bonadonna LV, Saunders MJ, Zegarra R, Evans C, Alegria-Flores K, Guio H. Why wait? The social determinants underlying tuberculosis diagnostic delay. PLoS One. 2017;12(9):e0185018.

18. Chakma B, Gomes D, Filipe PA, Soares P, de Sousa B, Nunes C. A temporal analysis on patient and health service delays in pulmonary tuberculosis in Portugal: inter and intra‑regional differences and in(equalities) between gender and age. BMC Public Health. 2022;22(1):1830.

19. Ribeiro RM, Havik PJ, Craveiro I. The circuits of healthcare: Understanding healthcare seeking behaviour-A qualitative study with tuberculosis patients in Lisbon, Portugal. PLoS One. 2021;16(12):e0261688.

20. Stjepanović M, Škodrić-Trifunović V, Radisavljević-Pavlović S, Roksandić-Milenković M, Milin-Lazović J, Babić U, et al. Patient, Healthcare System and Total Delay in Tuberculosis Diagnosis and Treatment Among Serbian Population. Acta Clin Croat. 2018;57(2):257-63.

21. Seid A, Metaferia Y. Factors associated with treatment delay among newly diagnosed tuberculosis patients in Dessie city and surroundings, Northern Central Ethiopia: a cross-sectional study. BMC Public Health. 2018;18(1):931.

22. Sagbakken M, Frich JC, Bjune GA. Perception and management of tuberculosis symptoms in Addis Ababa, Ethiopia. Qual Health Res. 2008;18(10):1356-66.

23. Kuznetsov VN, Grjibovski AM, Mariandyshev AO, Johansson E, Bjune GA. Two vicious circles contributing to a diagnostic delay for tuberculosis patients in Arkhangelsk. Emerg Health Threats J. 2014;7:24909.

24. PORDATA. População residente com 16 a 64 anos e 65 a 89 anos: por nível de escolaridade completo mais elevado (%). Available at <https://www.pordata.pt/portugal/populacao+residente+com+16+a+64+anos+e+65+a+89+anos+por+nivel+de+escolaridade+completo+mais+elevado+(percentagem)-2266> (Accessed on 10 February 2023).2023.

25. INE. Remuneração média mensal de base (€) por Localização geográfica (NUTS - 2013). Available at  <https://www.ine.pt/xportal/xmain?xpid=INE&xpgid=ine_indicadores&indOcorrCod=0006910&contexto=bd&selTab=tab2&xlang=PT> (Accessed on 10 February 2023)2022.

26. PORDATA. Taxa de desemprego: total e por grupo etário (%).  Available at <https://www.pordata.pt/Portugal/Taxa+de+desemprego+total+e+por+grupo+et%C3%A1rio+(percentagem)-553>  (Accessed on 1 March 2023)2023.

27. WHO. Alcohol. Available at  <https://www.who.int/news-room/fact-sheets/detail/alcohol>  (Accessed on 09 February 2023)2022.

28. Bonnechère B, Cissé K, Millogo T, Ouédraogo GH, Garanet F, Ouedraogo MA, et al. Tobacco use and associated risk factors in Burkina Faso: results from a population-based cross-sectional survey. BMC Public Health. 2019;19(1):1466.

29. Zubair F, Husnain MIU, Zhao T, Ahmad H, Khanam R. A gender-specific assessment of tobacco use risk factors: evidence from the latest Pakistan demographic and health survey. BMC Public Health. 2022;22(1):1133.

30. Luba TR, Tang S, Liu Q, Gebremedhin SA, Kisasi MD, Feng Z. Knowledge, attitude and associated factors towards tuberculosis in Lesotho: a population based study. BMC Infect Dis. 2019;19(1):96.

31. Vericat-Ferrer M, Ayala A, Ncogo P, Eyene-Acuresila J, García B, Benito A, et al. Knowledge, Attitudes, and Stigma: The Perceptions of Tuberculosis in Equatorial Guinea. Int J Environ Res Public Health. 2022;19(14).

32. Ranzani OT, Rodrigues LC, Waldman EA, Prina E, Carvalho CRR. Who are the patients with tuberculosis who are diagnosed in emergency facilities? An analysis of treatment outcomes in the state of São Paulo, Brazil. J Bras Pneumol. 2018;44(2):125-33.

33. Comissão para a Cidadania e a Igualdade de Género. Igualdade de Género em Portugal: Indicadores-chave 2017: Presidência do Conselho de Ministros; 2017.

34. PORDATA. Salário médio mensal dos trabalhadores por conta de outrem: remuneração base e ganho por sexo. Available at <https://www.pordata.pt/portugal/salario+medio+mensal+dos+trabalhadores+por+conta+de+outrem+remuneracao+base+e+ganho+por+sexo-894>. (Accessed on 10 February 2023).2023.

35. PORDATA. Taxa de desemprego: total e por sexo (%). Available at <https://www.pordata.pt/Portugal/Taxa+de+desemprego+total+e+por+sexo+(percentagem)-550> (Accessed on 10 February 2023).2023.

36. Chinwong D, Mookmanee N, Chongpornchai J, Chinwong S. A Comparison of Gender Differences in Smoking Behaviors, Intention to Quit, and Nicotine Dependence among Thai University Students. J Addict. 2018;2018:8081670.

37. Dalmau R. Women and tobacco, a gender perspective. e-Journal of Cardiology Practice. 2021;20.

38. Cai W, Wu F. Influence of Income Disparity on Child and Adolescent Education in China: A Literature Review. New Dir Child Adolesc Dev. 2019;2019(163):97-113.

39. Yang D, Zheng G, Wang H, Li M. Education, Income, and Happiness: Evidence From China. Front Public Health. 2022;10:855327.

40. PORDATA. Ganho médio mensal dos trabalhadores por conta de outrem: total e por nível de qualificação. Available at <https://www.pordata.pt/portugal/ganho+medio+mensal+dos+trabalhadores+por+conta+de+outrem+total+e+por+nivel+de+qualificacao-890> (Accessed on 10 February 2023).2023.

41. OECD. Unemployment rates by education level (indicator). doi: 10.1787/6183d527-en (Accessed on 09 February 2023)2023.

42. Dias P, Oliveira A, Lopes C. Social and behavioural determinants of alcohol consumption. Annals of Human Biology. 2011;38(3):333-44.

43. Latunji OO, Akinyemi OO. FACTORS INFLUENCING HEALTH-SEEKING BEHAVIOUR AMONG CIVIL SERVANTS IN IBADAN, NIGERIA. Ann Ib Postgrad Med. 2018;16(1):52-60.

44. Abuduxike G, Aşut Ö, Vaizoğlu SA, Cali S. Health-Seeking Behaviors and its Determinants: A Facility-Based Cross-Sectional Study in the Turkish Republic of Northern Cyprus. Int J Health Policy Manag. 2020;9(6):240-9.

45. Jurcev Savicevic A, Popovic-Grle S, Milovac S, Ivcevic I, Vukasovic M, Viali V, et al. Tuberculosis knowledge among patients in out-patient settings in Split, Croatia. Int J Tuberc Lung Dis. 2008;12(7):780-5.

46. Gautam N, Karki RR, Khanam R. Knowledge on tuberculosis and utilization of DOTS service by tuberculosis patients in Lalitpur District, Nepal. PLoS One. 2021;16(1):e0245686.

47. Harding A, Richardson S. Unemployment and income distribution. Discussion paper no. 32: National Centre for Social and Economic Modelling, Faculty of Management, University of Canberra; 1998.

48. Ormond G, Murphy R. The effect of alcohol consumption on household income in Ireland. Alcohol. 2016;56:39-49.

49. Huckle T, You RQ, Casswell S. Socio-economic status predicts drinking patterns but not alcohol-related consequences independently. Addiction. 2010;105(7):1192-202.

50. Huddart S, Bossuroy T, Pons V, Baral S, Pai M, Delavallade C. Knowledge about tuberculosis and infection prevention behavior: A nine city longitudinal study from India. PLoS One. 2018;13(10):e0206245.

51. Popovici I, French MT. Does Unemployment Lead to Greater Alcohol Consumption? Ind Relat (Berkeley). 2013;52(2):444-66.

52. Compton WM, Gfroerer J, Conway KP, Finger MS. Unemployment and substance outcomes in the United States 2002-2010. Drug Alcohol Depend. 2014;142:350-3.

53. Li C, Long C, Zhang M, Zhang L, Liu M, song M, et al. The influence of alcohol consumption on tobacco use among urban older adults: evidence from western China in 2017. Sustainability. 2022;14.

54. Jiang N, Lee YO, Ling PM. Association between tobacco and alcohol use among young adult bar patrons: a cross-sectional study in three cities. BMC Public Health. 2014;14:500.

55. Hoa NP, Thorson AE, Long NH, Diwan VK. Knowledge of tuberculosis and associated health-seeking behaviour among rural Vietnamese adults with a cough for at least three weeks. Scand J Public Health Suppl. 2003;62:59-65.

56. Senkoro M, Hinderaker SG, Mfinanga SG, Range N, Kamara DV, Egwaga S, et al. Health care-seeking behaviour among people with cough in Tanzania: findings from a tuberculosis prevalence survey. Int J Tuberc Lung Dis. 2015;19(6):640-6.
