# Supplementary material for: Investigating the role of symptom valorisation in tuberculosis patient delay in urban areas in Portugal
Source: BMC Public Health. 2023 Dec 5;23:2421. doi: 10.1186/s12889-023-17319-7 (PMC10696854; doi:10.1186/s12889-023-17319-7)
Supplement: Supplementary file 3 — Additional file 3: Supplementary Table 3. Comparing included and excluded subjects. Comparative analysis with hypothesis tests between individuals included and excluded of the study. [file 12889_2023_17319_MOESM3_ESM.docx]

Comparing included and excluded subjects

**Supplementary table 3 – Comparing included and excluded subjects.** Comparative analysis with hypothesis tests between individuals included and excluded of the study

|  | **Included in the study** | |  | |
| --- | --- | --- | --- | --- |
| **Variable** | **No**, N = 39^1^ | **Yes**, N = 75^1^ | **N** | **p-value**^2^ |
| **Age** |  |  | 114 | 0.054 |
| Median (IQR) | 45 (32.0, 54.5) | 50 (41.0, 60.0) |  |  |
| **Age categories** |  |  | 113 | 0.566 |
| 18 - 44 | 18 (47.4%) | 28 (37.3%) |  |  |
| 45 - 64 | 16 (42.1%) | 36 (48.0%) |  |  |
| 65+ | 4 (10.5%) | 11 (14.7%) |  |  |
| Unknown ^3^ | 1 | 0 |  |  |
| **Gender** |  |  | 114 | 0.847 |
| Men | 29 (74.4%) | 57 (76.0%) |  |  |
| Women | 10 (25.6%) | 18 (24.0%) |  |  |
| **City of residence** |  |  | 114 | 0.168 |
| Lisbon | 11 (28.2%) | 31 (41.3%) |  |  |
| Oporto | 28 (71.8%) | 44 (58.7%) |  |  |
| **Education** |  |  | 113 | 0.697 |
| 4th grade | 10 (25.6%) | 23 (31.1%) |  |  |
| 9th grade | 19 (48.7%) | 30 (40.5%) |  |  |
| Secondary/University | 10 (25.6%) | 21 (28.4%) |  |  |
| Unknown^3^ | 0 | 1 |  |  |
| **Unemployment** |  |  | 108 | 0.564 |
| No | 29 (82.9%) | 57 (78.1%) |  |  |
| Yes | 6 (17.1%) | 16 (21.9%) |  |  |
| Unknown^3^ | 4 | 2 |  |  |
| **Household income** |  |  | 90 | 0.148 |
| 650€ or less | 13 (44.8%) | 15 (24.6%) |  |  |
| 651-1000€ | 6 (20.7%) | 19 (31.1%) |  |  |
| More than 1000€ | 10 (34.5%) | 27 (44.3%) |  |  |
| Unknown^3^ | 10 | 14 |  |  |
| **Smoking habits** |  |  | 114 | 0.713 |
| No | 14 (35.9%) | 29 (38.7%) |  |  |
| Ex-smoker | 11 (28.2%) | 16 (21.3%) |  |  |
| Yes | 14 (35.9%) | 30 (40.0%) |  |  |
| **Alcohol consumption frequency** |  |  | 114 | 0.296 |
| Never | 14 (35.9%) | 37 (49.3%) |  |  |
| Sometimes | 8 (20.5%) | 9 (12.0%) |  |  |
| Regularly | 17 (43.6%) | 29 (38.7%) |  |  |
| **First initiative addressing symptoms** |  |  | 99 | 0.275 |
| Calling the emergency line | 0 (0.0%) | 1 (1.4%) |  |  |
| Contacting a doctor outside the formal health system | 1 (3.7%) | 1 (1.4%) |  |  |
| Going to the doctor | 25 (92.6%) | 66 (91.7%) |  |  |
| Self-medicating | 0 (0.0%) | 4 (5.6%) |  |  |
| Contact a pharmacist | 1 (3.7%) | 0 (0.0%) |  |  |
| Unknown^3^ | 12 | 3 |  |  |
| **Unit of the first appointment** |  |  | 112 | **0.002** |
| Emergency services | 9 (24.3%) | 35 (46.7%) |  |  |
| Hospital | 17 (45.9%) | 12 (16.0%) |  |  |
| Primary health care | 11 (29.7%) | 28 (37.3%) |  |  |
| Unknown^3^ | 2 | 0 |  |  |
| **Number of symptoms** |  |  | 114 | **<0.001** |
| Mean (SD) | 2.0 (2.1) | 3.8 (1.5) |  |  |
| **Knowledge level about tuberculosis (0 - 5)** |  |  | 114 | 0.249 |
| Median (IQR) | 4.0 (3.0, 5.0) | 4.0 (3.0, 5.0) |  |  |
| **Symptom valorisation** |  |  | 109 | **<0.001** |
| No | 1 (2.9%) | 29 (38.7%) |  |  |
| Yes | 33 (97.1%) | 46 (61.3%) |  |  |
| Unknown^3^ | 5 | 0 |  |  |
| **Patient diagnosis delay** |  |  | 94 | **<0.001** |
| Median (IQR) | 0.0 (0.0, 8.5) | 25.0 (11.5, 63.5) |  |  |
| Unknown^3^ | 20 | 0 |  |  |
| **Patient delay categorised on 21 days** |  |  | 94 | **0.006** |
| Delayed | 4 (21.1%) | 42 (56.0%) |  |  |
| Not delayed | 15 (78.9%) | 33 (44.0%) |  |  |
| Unknown^3^ |  |  |  |  |
| **Patient delay categorised on 30 days** |  |  | 94 | 0.083 |
| Delayed | 4 (21.1%) | 32 (42.7%) |  |  |
| Not delayed | 15 (78.9%) | 43 (57.3%) |  |  |
| Unknown^3^ | 20 | 0 |  |  |
| ^1^n (%) | | | | |
| ^2^Wilcoxon rank sum test; Pearson's Chi-squared test; Fisher's exact test.  ^3^Unknown values were not considered in the calculus of the percentages. | | | | |
